# Differences in Ovarian and Other Cancers Risks by Population and *BRCA* Mutation Location

**DOI:** 10.3390/genes12071050

**Published:** 2021-07-08

**Authors:** Masayuki Sekine, Koji Nishino, Takayuki Enomoto

**Affiliations:** Department of Obstetrics and Gynecology, Niigata University Graduate School of Medical and Dental Sciences, Niigata 951-8510, Japan; knishino@med.niigata-u.ac.jp (K.N.); enomoto@med.niigata-u.ac.jp (T.E.)

**Keywords:** *BRCA1/2*, hereditary breast and ovarian cancer, *BRCA*-related cancer, risk-reducing salpingo-oophorectomy

## Abstract

Hereditary breast and ovarian cancer is caused by a germline mutation in *BRCA1* or *BRCA2* genes. The frequency of germline *BRCA1/2* gene mutation carriers and the ratio of germline *BRCA1* to *BRCA2* mutations in *BRCA*-related cancer patients vary depending on the population. Genotype and phenotype correlations have been reported in *BRCA* mutant families, however, the correlations are rarely used for individual risk assessment and management. *BRCA* genetic testing has become a companion diagnostic for PARP inhibitors, and the number of families with germline *BRCA* mutation identified is growing rapidly. Therefore, it is expected that analysis of the risk of developing cancer will be possible in a large number of *BRCA* mutant carriers, and there is a possibility that personal and precision medicine for the carriers with specific common founder mutations will be realized. In this review, we investigated the association of ovarian cancer risk and *BRCA* mutation location, and differences of other *BRCA*-related cancer risks by *BRCA1/2* mutation, and furthermore, we discussed the difference in the prevalence of germline *BRCA* mutation in ovarian cancer patients. As a result, although there are various discussions, there appear to be differences in ovarian cancer risk by population and *BRCA* mutation location. If it becomes possible to estimate the risk of developing BRCA-related cancer for each *BRCA* mutation type, the age at risk-reducing salpingo-oophorectomy can be determined individually. The decision would bring great benefits to young women with germline *BRCA* mutations.

## 1. Introduction

The *BRCA1* gene, located on chromosome 17, and the *BRCA2* gene, located on chromosome 13, are involved in the repair of double-strand DNA breaks and cell-cycle checkpoints in response to DNA damage. The functions of two genes preserve genomic stability as tumor suppressor genes [1,2,3,4]. The overall prevalence of germline mutations in *BRCA1* and *BRCA2* genes in unaffected women has been estimated at 0.11% and 0.24%, respectively [5]. So far, many mutations have been identified in *BRCA1/2* genes. Among them, the same mutation has been found in multiple, unrelated families and can be traced back to a common ancestor. Such mutations are so-called common founder mutations observed in specific populations, e.g., 187delAG and 5385insC of *BRCA1* and the 6174delT of *BRCA2* in the Ashkenazi Jewish and L63X and Q934X of *BRCA1* in a Japanese population [6,7,8,9]. Certain common founder mutations have also been identified in other populations [10,11,12,13,14,15,16]. The carriers of *BRCA1/2* mutations have a high risk of specific cancer, such as breast, ovarian, pancreatic, and prostate cancer. However, the probability of cancer development in the carriers is variable, even within families with the same variant [17,18,19]. It is still unknown whether the risk of developing cancer in the carriers is related only to the specific mutation or whether additional genetic and environmental factors exist.

In this review article, we used three databases (PubMed, Google Scholar, and Web of Science) and references or related articles to conduct a review of the cancer risk by *BRCA* mutation types. We identified articles in the databases using the following search string: (“ovarian cancer” OR “breast cancer” OR “common mutation” OR “founder mutation” OR “cancer risk” OR “ethnicity” OR “race” OR “population”) AND “*BRCA*”. Given the search results, we added the words “Prostate cancer,” “Pancreatic cancer,” “Melanoma,” “risk-reducing salpingo-oophorectomy,” and “risk-reducing mastectomy” to cover all relevant articles.

## 2. Differences of the Prevalence of Germline *BRCA* Mutation in Ovarian Cancer Patients

### 2.1. Prevalence of Germline BRCA1/2 Mutation in Ovarian Cancer Patients

The risk of developing ovarian cancer is thought to increase with early menarche, delayed menopause, nulliparity, infertility, and obesity, however, the strongest risk factor for ovarian cancer is a positive family history of breast and/or ovarian cancer [20,21,22]. The risk of developing ovarian cancer is 2 to 6 times higher in those who have breast cancer or ovarian cancer as first-degree relatives [23,24,25]. Hereditary ovarian cancer occurs as part of a hereditary tumor represented by Hereditary breast and ovarian cancer (HBOC) and Lynch syndrome. Of these, HBOC is the most involved and is estimated to account for about 65–85% of hereditary ovarian cancers [26]. In large-scale epidemiologic studies, the penetrance of the *BRCA1/2* gene for female breast cancer was about 70%, and there was almost no difference between *BRCA1* and *BRCA2* [27]. However, the penetrance of the *BRCA1* or *BRCA2* gene for ovarian cancer has been reported to be about 40% and 20%, respectively [27]. On the other hand, the risk of developing male breast cancer, prostate cancer, pancreatic cancer, and melanoma in *BRCA2* mutation carriers has been reported to be higher than in *BRCA1* [26]. Given the genetic risk of developing ovarian cancer in a population, the frequency of *BRCA* gene carriers in the general population leads to the direct estimation of risk factors. In addition, the frequency of germline *BRCA1/2* gene mutation carriers and the ratio of germline *BRCA1* to *BRCA2* mutations in ovarian cancer patients may vary depending on the population.

Table 1 shows the differences in germline BRCA1/2 mutation prevalence between population and country in ovarian cancer patients [28,29,30,31,32,33,34,35,36,37,38]. The frequency of germline *BRCA* mutation ranged from 5% to 30%. Among these reports, the high frequency of germline *BRCA* mutation in Ashkenazi Jews stands out as already reported [22]. The frequency of germline *BRCA* mutation in the USA, Canada, Australia, and Japan showed average values of about 15% [29,30,35,38]. On the other hand, the frequency of germline *BRCA* mutation varies in European countries, but that of Finland, Sweden, Denmark and Iceland appear to be relatively low [31,32,33,39]. The ratio of *BRCA1* to *BRCA2* mutations varies from population to population, but it is consistent with previous reports that germline *BRCA1* mutation was more common than germline *BRCA2* mutation in ovarian cancer cases. However, reports from Iceland and Poland show that germline *BRCA2* mutation was more frequent than germline *BRCA1* mutation [39,40]. The exact reason for this event is unknown, but the presence and spread of common founder mutations among ethnically different populations may have affected the proportion of germline *BRCA1/2* mutation.

### 2.2. Histological Subtypes in BRCA-Related Ovarian Cancer

In many mutational analyses of *BRCA1/2* genes for epithelial ovarian cancer, we picked up large-scale epidemiological studies of more than 500 ovarian cancer patients. Table 2 shows that the rate of germline *BRCA* mutation of each histological type varies by the country where the study was conducted [28,29,30,34,35,36,37,38]. Regarding high-grade serous carcinoma, the mutation rate showed a range of 16% to 28%, and the rate tended to be higher in Asian countries than in Western countries. Almost 20% of Low-grade serous carcinoma in Japan or Korea were *BRCA* mutated, while this was only 6% in the USA or German population. Although it is not clear due to the small number of cases, the frequency of *BRCA* mutation in Low-grade serous carcinoma may be lower in Asia than that in European countries. Regarding mucinous carcinoma, no case with germline *BRCA* mutation was found in Western countries and Japan, however, germline *BRCA1/2* mutation was found in 4 of 57 cases (7.0%) in China and 1 of 18 cases (5.6%) in South Korea. There is no doubt that patients with mucinous carcinoma rarely have germline *BRCA* mutation [8], but it is unclear whether the involvement of *BRCA* gene mutations in the pathogenic mechanism of mucinous carcinoma differs between Western and Asian countries. In the histological diagnosis of ovarian cancer, the existence of a mixed type is also known, and it may be related to the diversity of pathological diagnosis rather than the molecular biological reason. Regarding endometrioid carcinoma, the mutation rate was the lowest in Japan at 6.7% and the highest in Germany and South Korea at 13.0%, but there seems to be no clear difference between the countries. There are few reports on the new classification, Seromucinous tumor of the ovary, but in the analysis of four cases in Japan and seven cases in South Korea, no case with germline *BRCA1/2* mutation was found in the tumor.

Regarding clear cell carcinoma, Germany had the lowest rate at 0%, followed by Japan at 2.1%, and other countries at about 7%. There are significant differences between Western and East Asian countries regarding the frequency of clear cell carcinoma in all types of epithelial ovarian cancer. For example, the frequency of clear cell carcinoma in the United States is about 6%, but the frequency of clear cell carcinoma in Japan is about 25%, which is a four-fold difference [45,46]. It is known that the incidence of endometriosis is high in East Asia [47], and it is presumed that this is a factor in the higher frequency of clear cell carcinoma that develops from endometriosis than in Western countries [48]. Especially in Japan, there is a tendency that drug therapy with GnRH agonists and Dienogest is preferred over surgical therapy as a treatment for endometriosis [49], so there is a relatively high possibility that clear cell carcinoma will develop from an endometriotic cyst.

Regarding the difference between *BRCA1* and *BRCA2* in the frequency of histological types in each country, the frequency of serous carcinoma in cases with *BRCA1* or *BRCA2* mutation was about 80%, and there was almost no difference between *BRCA1* and *BRCA2*. Among ovarian cancer patients in China and South Korea, five cases of mucinous carcinoma with *BRCA1/2* mutation were found, of which three cases had a *BRCA1* mutation and two cases had a *BRCA2* mutation.

## 3. Association of Breast/Ovarian Cancer Risk and *BRCA* Mutation Location

Genotype and phenotype correlations have been reported in *BRCA* mutant families. At present, such correlations are rarely used for individual risk assessment and management. However, the data of the mutant carriers have accumulated dramatically, so the genotype and phenotype correlations may be utilized for individual risk assessment.

The ovarian cancer cluster region (OCCR) was identified in or near exon 11 in both the *BRCA1* and *BRCA2* genes. Mutations within the lesion increase the ratio of ovarian cancer to breast cancer, unlike variants elsewhere in both genes. The Consortium of Investigators of Modifiers of *BRCA1/2* (CIMBA) revealed that the incidence of ovarian cancer is high in patients with germline *BRCA* mutation in the OCCR in about 30,000 *BRCA* mutant carriers in 33 countries around the world [50]. These results are consistent with prior reports of OCCR in *BRCA1/2* genes [51,52,53]. On the other hand, regarding the breast cancer cluster region (BCCR), multiple regions other than exon 11 have been reported for both genes [50,54]. Rebbeck et al. speculated why OCCR is present in the *BRCA1* gene as follows. Mutations in exon 11 could produce a partial *BRCA1* protein encoded by the known exon 11 splice variant, while the full-length protein is lost by the process of nonsense-mediated mRNA decay (NMD). Thus, it is biologically plausible that individuals carrying mutations within exon 11 (and the OCCR) may have a different phenotype than other mutations for *BRCA1* [50].

**Figure 1:**
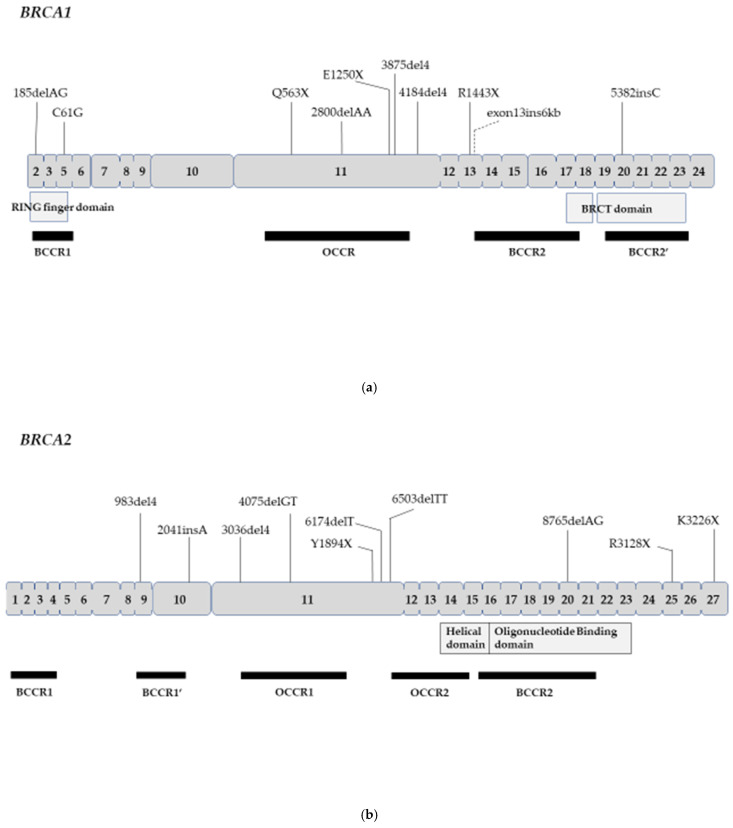
Prevalence of (**a**) *BRCA1* and (**b**) *BRCA2* common founder mutations by location. Putative functional domains are shown in the middle. Regions inferred to be breast cancer cluster region (BCCR) and ovarian cancer cluster region (OCCR) are shown at the bottom.

The Breast Cancer Information Core (BIC) database contains DNA sequence variations reported from around the world. The total number of database entries was 15,311 and 14,914 in *BRCA1* and *BRCA2*, respectively [55]. Table 3 shows the top 10 pathogenic mutations in *BRCA1* or *BRCA2*. Regarding the judgment of clinical pathogenic importance, according to the opinion of the BIC steering committee, the sequence change of this type interferes with gene function and results in an increased risk of cancer based on available data [55]. For the *BRCA1* gene, the most frequently reported mutation is 185delAG, followed by 5382insC, C61G, 4184del4, R1443X, 3875del4, and exon13ins6kb. The above seven variants showed the number of entries of 100 times or more. Among these common founder mutations, the mutations located within OCCR are Q563X, 2800delAA, E1250X, and 3875del4 in *BRCA1* and 4075delGT in *BRCA2* (Figure 1). In a recent report, Yoshihara et al. reported that more than 50% of Japanese ovarian cancer patients with *BRCA1* or *BRCA2* mutations were within the OCCR after excluding 16 cases with L63X founder mutation [56]. On the other hand, Cardoso et al. reported that 33% (20/60) of Argentine ovarian cancer patients with *BRCA1* mutations were beyond the OCCR, in stark contrast with 61% (22/36) of the patients with *BRCA2* mutations being inside the OCCR [57]. Moreover, Teixeira et al. reported that among Dutch *BRCA1* families, ovarian cancer risks were higher in women with OCCR mutations than non-OCCR mutations, but not in *BRCA2* families [58].

Rebbeck et al. reported that exon 11 mutations were associated with earlier ages in breast and ovarian cancer diagnosis and mutations conferring NMD or premature termination codon were associated with a later age at breast cancer diagnosis in *BRCA1*. In *BRCA2*, the mean age was greater for mutations in OCCR vs. mutations not in OCCR (45.0 vs. 43.9 years, *p* < 0.001), lower for mutations in BCCR1 vs. mutations not in BCCR1 (42.6 vs. 44.3 years; *p* = 0.004), and lower for mutations in BCCR2 vs. mutations not in BCCR2 (43.5 vs. 44.3 years, *p* = 0.04) [50].

Various common founder mutations of the *BRCA* gene have been reported by different populations in the world [28,59]. It has been reported that some of these mutation carriers have a different risk of developing cancer than the overall mutation carriers. For example, Satagopan et al. reported that the estimated lifetime ovarian cancer risks were 66% (95% CI, 37–100%) due to 185delAG mutation and 29% (95% CI, 16–69%) due to 5382insC mutation [60]. There is also a report that the presence of 5382insC decreased and C61G in *BRCA1* increased the risk of peritoneal cancer (*p* = 0.049 vs. *p* = 0.013) in the Polish population who underwent risk-reducing salpingo-oophorectomy (RRSO) [61]. Ashkenazi Jewish families with the 6174delT founder mutation were more likely to have a family member with ovarian cancer (OR = 1.58; *p* = 0.002) [62]. Breast cancer risks for carriers of 6174delT were lower than those of all *BRCA1* carriers (43% by age 70, 95% CI, 14% to 62%; *p* = 0.007 compared with all *BRCA1* mutation carriers), on the other hand, the ovarian cancer risks in the carriers were somewhat higher than the average *BRCA2* risks (20% vs. 11%) [63]. The corresponding ovarian cancer risks were 14% (95% CI, 2% to 24%), 33% (8% to 50%), and 20% (2% to 35%) in carriers of the 185delAG, 5382insC in *BRCA1* and 6174delT mutations in *BRCA2*, respectively [63]. The K3326X mutation was associated with increased risk of breast cancer (OR = 1.28, 95% CI = 1.17 to 1.40) independent of additional *BRCA2* mutations and demonstrated strong association with serous ovarian cancer (OR = 1.46, 95% CI = 1.2 to 1.70), but not with prostate cancer [64].

We found L63X and Q934X as Japanese common founder mutations previously [8]. The clinical characteristics (e.g., subtype and nuclear grade of resultant cancer) of breast cancer patients with L63X might differ from those in patients with other *BRCA* mutations, however, the elevation of ovarian or breast cancer risk was not detected [65]. After excluding L63X founder mutation, the proportion of patients with a family history of ovarian cancer and germline *BRCA1* mutations outside the OCCR was lower and the proportion of patients with a family history of breast cancer and germline *BRCA1* mutations within the OCCR was relatively lower [56]. There are, relatively, many reports that *BRCA* carriers with common founder mutations have different risks of developing breast and ovarian cancer compared with the overall *BRCA1/2* mutation, however, the results of only a few common founder mutations have been validated by multiple studies. Since the frequency of specific common founder mutations in each population varies, so does the number of breast and ovarian cancer patients who carry the mutation. In other words, 185delAG in the Ashkenazi Jewish population can be analyzed in many breast and ovarian cancer cases, so it is possible to analyze the risk of developing cancer relatively easily. However, sufficient statistical power is often not obtained in the analysis of other common founder mutations. *BRCA* genetic testing has become a companion diagnostic for PARP inhibitors, and the number of families with germline *BRCA* mutation identified is growing rapidly [66]. Therefore, it is expected that analysis of the risk of developing cancer will be possible in a large number of mutant carriers, and there is a possibility that personal and precision medicine for carriers with specific common founder mutations will be realized [67].

## 4. Differences of other *BRCA*-Related Cancers Risks by *BRCA1/2* Mutation

### 4.1. Contralateral Breast Cancer Risk

Ten-year cumulative contralateral Breast Cancer (CBC) risks were 21.1% for *BRCA1*, 10.8% for *BRCA2* mutation carriers and 5.1% for non-carriers [68]. On the other hand, the 15-year actuarial risk of CBC was 36.1% for *BRCA1* carriers and was 28.5% for *BRCA2* carriers [69]. The average cumulative risks by age 70 years for *BRCA1* and *BRCA2* carriers were estimated to be 83% and 62% for CBC [70,71].

### 4.2. Male Breast Cancer

Tai et al. reported that the cumulative risks of male breast cancer were higher in both *BRCA1* and *BRCA2* carriers than in non-carriers at all ages. The relative risks of developing breast cancer were highest for men in their 30s and 40s. Both the relative and cumulative risks were higher for *BRCA2* carriers than for *BRCA1* carriers. The estimated cumulative risk of breast carcinoma for male *BRCA1* mutation carriers at age 70 years was 1.2% and for *BRCA2* mutation carriers, 6.8% [72]. In addition, both retrospective and prospective analyses confirmed that breast cancer risk in men was 7.1% by age 70 years and 8.4% by age 80 years in *BRCA2* carriers [71,73]. Struewing et al. reported that four (3.6%) and fifteen (13.6%) of 110 Israeli Jewish male breast cancer patients carried the *BRCA1* 185delAG and *BRCA2* 6174delT founder mutation, respectively, but not *BRCA1* 5382insC [74]. Lubinski et al. reported that a high risk of male breast cancer was observed with the *BRCA2* 6503delTT mutation (OR = 15.7; *p* = 0.023) [62].

### 4.3. Prostate Cancer

Based on previously estimated population frequencies of *BRCA1* and *BRCA2* mutations, it was estimated that *BRCA1* mutations confer a relative risk of prostate cancer of approximately 3.7-fold and 8.6-fold, which translates to an 8.6% and 15% cumulative risk by age 65 years [71,75,76]. A recent meta-analysis revealed that the relative risk of prostate cancer is 1.35-fold and 2.64-fold in *BRCA1* and *BRCA2* carriers, respectively. Overall survival was significantly worse among germline *BRCA2* carriers compared to non-carriers [77]. *BRCA2*-related prostate cancer has been associated with a higher histologic grade and results in a poorer overall survival [78,79]. It was reported that *BRCA2*, in particular, confers a more aggressive phenotype with a higher probability of locally advanced and metastatic disease, and should be considered a prognostic marker associated with poorer survival [80]. Agalliu et al. reported that *BRCA2* mutation confers a 3-fold elevated risk of high-grade prostate cancer. Although *BRCA1* mutations were not associated with prostate cancer, the *BRCA1* 185delAG was associated with high Gleason score tumors [81].

### 4.4. Pancreatic Cancer

Several studies reported that *BRCA2* carriers had higher relative and cumulative risks of pancreatic cancer compared to *BRCA1* carriers [71,82,83]. Recently, Mocci et al. reported that *BRCA1* carriers were at increased risk of pancreatic cancer [standardized incidence ratios (SIR) = 4.11] as were *BRCA2* carriers (SIR = 5.79) in a retrospective cohort analysis [84]. A prospective study of 5,149 females with *BRCA1* or *BRCA2* carriers showed a significant 2.4-fold increase in the incidence of pancreatic cancer and the increase in the incidence of pancreatic cancer was similar for *BRCA1* (SIR = 2.55) and *BRCA2* (SIR = 2.13) [85]. Among unselected pancreatic cancer patient cohorts, multiple studies have shown to estimate the incidence of germline *BRCA* mutations ranged from 0.7–5.7% for BRCA2 and 0.3–2.3% for BRCA1 [86]. Of the 145 Jewish pancreatic adenocarcinoma patients, 8 patients (5.5%) were found to have *BRCA* mutations, 6 patients (4.1%) carried a *BRCA2* mutation (6174delT) and 2 patients (1.3%) carried a *BRCA1* mutation (185delAG and 5382insC) [87].

### 4.5. Melanoma

A few studies suggest that melanoma risk, both cutaneous and ocular, may be elevated in some families with *BRCA2* carriers [71,88]. Moran et al. reported that an increased risk for ocular melanoma in *BRCA2* carriers in 490 *BRCA*-mutated families [89].

### 4.6. Endometrial Cancer

Previous studies suggested that endometrial serous adenocarcinoma is not *BRCA*-related cancer and is more associated with tamoxifen exposure than with the effects of germline *BRCA* mutations [90,91]. Recently, in a prospective cohort study of *BRCA* carriers who received only RRSO, not hysterectomy, endometrial cancer developed in eight patients in a median follow-up of 5.1 years, with no apparent increased risk after RRSO, on the other hand, *BRCA1* carriers had an increased risk of endometrial serous adenocarcinoma [92]. Furthermore, a large Dutch nationwide cohort study revealed that *BRCA1/2* carriers have a 2- to 3-fold increased risk for endometrial cancer, with the highest risk observed for the rare subgroups of serous-like and p53-abnormal endometrial cancer in *BRCA1* carriers [93].

## 5. Conclusions

Although there are various discussions, there appear to be differences in ovarian cancer risk by ethnicity and *BRCA* mutation types. These mutation-specific risks coincide with known or hypothesized functional domains and provide a basis around which accurate risk estimates can be generated for women who have inherited a particular *BRCA1/2* mutation. While RRSO is certain to reduce the risk of ovarian cancer, there are some concerns about reducing the risk of breast cancer. In a prospective cohort study, Kauff et al. reported that RRSO did not significantly reduce the breast cancer risk in *BRCA1* carriers [94]. However, the latest meta-analysis has shown a significant reduction in breast cancer risk and overall mortality rate, regardless of the past history of breast cancer [95]. The risk of breast cancer does not appear to be different between *BRCA1* and *BRCA2* carriers [95].

The National Comprehensive Cancer Network (NCCN) guidelines state that the age at RRSO should be based on the earlier age at diagnosis of ovarian cancer patients in the family [26]. If it becomes possible to estimate the risk of developing breast and ovarian cancer and the age of onset disease for each *BRCA* mutation type, the age at RRSO can be determined individually. Solsky et al. reported that for *BRCA1/2* carriers who delayed RRSO or who were identified with a mutation later in life, the OCCR mutation tended to be associated with lower life expectancy estimates than the BCCR and non-BCCR/OCCR mutations, so *BRCA1/2* cluster regions may provide more precise estimates of life expectancy in counseling and shared decision-making [54]. The decision would bring great benefits to young women with *BRCA* mutations, so we hope that a lot of verifiable research will be undertaken.

## Figures and Tables

**Table 1:** Differences of *BRCA1/2* mutation prevalence between race and country in ovarian cancer patients.

Country/Population	No. of Cases	*BRCA1*	*BRCA2*	Ratio
				*BRCA1:BRCA2*
Ashkenazi Jews [22]	840	182 (21.7%)	64 (7.6%)	2.8:1
USA [29]	1915	182 (8.5%)	98 (6.3%)	1.9:1
Canada [30]	977	75 (7.7%)	54 (5.5%)	1.4:1
Finland [31]	233	11 (4.7%)	2 (0.9%)	5.5:1
Sweden [32]	161	12 (7.5%)	1 (0.6%)	12:1
Denmark [33]	445	22 (4.9%)	4 (0.9%)	5.5:1
Iceland [39]	179	2 (1.1%)	10 (5.6%)	0.5:1
Poland [40]	309	23 (7.4%)	29 (9.4%)	0.8:1
Germany [34]	523	81 (15.5%)	28 (5.4%)	2.9:1
India [41]	239	37 (15.5%)	14 (5.9%)	2.6:1
Turkey [42]	102	10 (9.8%)	7 (6.9%)	1.4:1
Pakistan [43]	120	16 (13.3%)	3 (2.5%)	5.3:1
Colombia [44]	100	13 (13.0%)	2 (0.2%)	6.5:1
Australia [38]	809	70 (8.7%)	39 (4.8%)	1.8:1
Japan [35]	634	63 (9.9%)	30 (4.7%)	2.1:1
China [36]	1331	228 (17.1%)	70 (5.3%)	3.3:1
Korea [37]	805	106 (13.2%)	51 (6.3%)	2.1:1

**Table 2:** Prevalence of germline BRCA mutation by histological type in ovarian cancer patients.

Histological Classification	USA [29] (*n* = 1699)	Australia [38] (*n* = 891)	Germany [34] (*n* = 462)	Japan [35] (*n* = 609)	China [36] (*n* = 1044)	Korea [37] (*n* = 591)
High-grade serous	16.0% (240/1498)	16.6% * (118/709)	23.2% (94/406)	28.5% (78/274)	27.2% * (229/843)	22.3% (95/426)
Low-grade serous	5.7% (4/70)	N/A *	5.6% (1/18)	20.0% (1/5)	N/A *	19.4% (6/31)
Endometrioid	10.9% (7/64)	8.4% (10/119)	13.0% (3/23)	6.7% (8/120)	10.8% (7/65)	13.0% (7/54)
Clear cell	6.9% (4/58)	6.3% (4/63)	0.0% (0/6)	2.1% (4/187)	7.6% (6/79)	7.3% (4/55)
Mucinous	0.0% (0/9)	N/A	0.0% (0/9)	0.0% (0/19)	7.0% (4/57)	5.6% (1/18)
Seromucinous	N/A	N/A	N/A	0.0% (0/4)	N/A	0.0% (0/7)

N/A: not applicable; * including either grade.

**Table 3:** Common mutation types of *BRCA1* or *BRCA2* genes in the BIC database.

BIC Designation	Number of Entries	Exon	HGVS cDNA	HGVS Protein	Mutation Type	Population
***BRCA1***						
185delAG	2038	2	c.66_67delAG	p.Glu23ValfsTer17	Frameshift	Ashkenazi Jewish
5382insC	1093	20	c.5263_5264insC	p.Gln1756ProfsTer74	Frameshift	Ashkenazi Jewish
C61G	239	5	c.181T>G	p.Cys61Gly	Missense	Europe
4184del4	144	11	c.4065_4068delTCAA	p.Asn1355LysfsTer10	Frameshift	Asia
R1443X	136	13	c.4327C>T	p.Arg1443Ter	Nonsense	Europe
3875del4	124	11	c.3756_3759delGTCT	p.Ser1253ArgfsTer10	Frameshift	Europe
exon13ins6kb	111	13	N/A	N/A	Frameshift	N/A
E1250X	98	11	c.3748G>T	p.Glu1250Ter	Nonsense	Europe/Americas
Q563X	94	11	c.1687C>T	p.Gln563Ter	Nonsense	N/A
2800delAA	81	11	c.2681_2682delAA	p.Lys894ThrfsTer8	Frameshift	Europe
***BRCA2***						
6174delT	1093	11	c.5946_5946delT	p.Ser1982ArgfsTer22	Frameshift	Ashkenazi Jewish
K3326X	301	27	c.9976A>T	p.Lys3326Ter	Nonsense	N/A
3036del4	111	11	c.2808_2811delACAA	p.Ala938ProfsTer21	Frameshift	Americas
6503delTT	95	11	c.6275_6276delTT	p.Leu2092ProfsTer7	Frameshift	Americas/Europe
8765delAG	76	20	c.8537_8538delAG	p.Glu2846GlyfsTer22	Frameshift	Americas/Europe
2041insA	75	10	c.1813_1814insA	p.Asp605GlufsTer2	Frameshift	Europe
4075delGT	64	11	c.3847_3848delGT	p.Val1283LysfsTer2	Frameshift	N/A
Y1894X	62	11	c.5682C>G	p.Tyr1894Ter	Nonsense	N/A
983del4	61	9	c.755_758delACAG	p.Asp252ValfsTer24	Frameshift	N/A
R3128X	50	25	c.9382C>T	p.Arg3128Ter	Nonsense	Europe

N/A: not applicable.

## Data Availability

No data are available for this review article.
